# An open-source framework for neuroscience metadata management applied to digital reconstructions of neuronal morphology

**DOI:** 10.1186/s40708-020-00103-3

**Published:** 2020-03-26

**Authors:** Kayvan Bijari, Masood A. Akram, Giorgio A. Ascoli

**Affiliations:** Krasnow Institute for Advanced Study, George Mason University, Fairfax, VA USA

**Keywords:** Neuroscience curation, Metadata extraction, Knowledge engineering, Data sharing, Information management tools, Neuronal morphology

## Abstract

Research advancements in neuroscience entail the production of a substantial amount of data requiring interpretation, analysis, and integration. The complexity and diversity of neuroscience data necessitate the development of specialized databases and associated standards and protocols. NeuroMorpho.Org is an online repository of over one hundred thousand digitally reconstructed neurons and glia shared by hundreds of laboratories worldwide. Every entry of this public resource is associated with essential metadata describing animal species, anatomical region, cell type, experimental condition, and additional information relevant to contextualize the morphological content. Until recently, the lack of a user-friendly, structured metadata annotation system relying on standardized terminologies constituted a major hindrance in this effort, limiting the data release pace. Over the past 2 years, we have transitioned the original spreadsheet-based metadata annotation system of NeuroMorpho.Org to a custom-developed, robust, web-based framework for extracting, structuring, and managing neuroscience information. Here we release the metadata portal publicly and explain its functionality to enable usage by data contributors. This framework facilitates metadata annotation, improves terminology management, and accelerates data sharing. Moreover, its open-source development provides the opportunity of adapting and extending the code base to other related research projects with similar requirements. This metadata portal is a beneficial web companion to NeuroMorpho.Org which saves time, reduces errors, and aims to minimize the barrier for direct knowledge sharing by domain experts. The underlying framework can be progressively augmented with the integration of increasingly autonomous machine intelligence components.

## Introduction

Neuroscience is continuously producing an immense amount of complex and highly heterogeneous data typically associated with peer-reviewed publications. When building data-driven models of brain function, computational neuroscientists must engage in the laborious task of reviewing, annotating, and deriving many parameters required for numerical simulations. More generally, the process of curation consists of extracting, maintaining, and adding value to digital information from the literature and underlying datasets [6]. Mature reference management tools exist to aid general-purpose bibliography organization and content annotation, including Zotero [35], Mendeley [40], and EndNote [1]. Moreover, community-sourced terminologies [11, 14, 21, 38] and domain-specific markup languages [16, 24, 18] provide human-interpretable controlled vocabularies and machine-readable file formats, respectively. Efforts are also underway to generate standardized data models [15, 39, 36] and to formalize related concepts into robust ontologies [20, 23, 25]. As a result, full-text information retrieval systems are becoming indispensable research aids [13, 22, 28, 29].

Despite promising progress, neuroscience and related fields lacked until recently a user-friendly tool to annotate a dataset or journal article across a customizable variety of fields with a set of controlled vocabularies. At the same time, a systematic and well-documented extraction process is essential to keep the curated metadata updated over time and portable between different projects [32]. Perhaps the sole example of an open-source, web-based framework for the acquisition, storage, search, and reuse of scientific metadata is the CEDAR workbench [17]. On the one hand, the entirety of neuroscience is too broad and diverse to fully benefit from an all-encompassing metadata annotation tool. On the other, the most useful motivating applications are typically task specific and, consequently, difficult to compare with other developed tools. Meanwhile, several fundamental metadata dimensions, including details about the animal subject, the location within the nervous system, and the experimental condition, are largely common to even considerably distinct subfields of neuroscience. One possible approach is therefore to design a practical solution to a specific problem of interest while adhering to a strictly open-source implementation that may foster broad adoption and custom adaptation throughout the neuroscience community.

Here, we introduce a resource developed to promote and facilitate data sharing and metadata annotation for NeuroMorpho.Org, a repository providing unrestricted access to digital reconstructions of neuronal and glial morphology [2, 3]. The acquisition and release of morphological tracings begin with the continuous identification of newly published scientific reports describing data of interest [19, 26]. To annotate the reconstructions with proper metadata, the repository administrators have also been inviting data contributors to provide suitable information through a semi-structured Excel spreadsheet [33]. While the ecosystem of neuronal reconstructions has coalesced around a simple data standard for over two decades [30], selection and interpretation of metadata concepts remain highly variable and inconsistent. Thus, for every new dataset, a team of trained curators must validate or reconcile the author-provided information, complemented as needed by the associated publication, with the metadata schema and preferred nomenclature of the database. Many data releases also introduce new metadata concepts, which need to be integrated into the existing ontology and require updating relevant database hierarchies with appropriate terms. Although the described process is time-consuming, labor-intensive, and error-prone, metadata annotation is instrumental to enable NeuroMorpho.Org semantic queries [34] and machine accessibility through Application Programming Interfaces [4].

This article presents the NeuroMorpho.Org metadata portal, a novel, open-source, web-based tool for the efficient annotation and collaborative management of data descriptors for digital reconstructions of neuronal and glial morphology. The main goal of this effort is the gradual automation of the metadata extraction process to reduce the burden on database curators, thus streamlining the data release workflow for the benefit of the entire research community. A related motivation is to bring domain expertise closer to the crucial task of metadata curation by empowering data contributors with direct dataset annotation through a graphical user interface. The longer-term vision is to lay the training data foundation for augmenting neuro-curation with semi-autonomous machine learning components such as recommendation systems or natural language processing tools [8, 9, 12]. With this report, we freely release the documented code base to date and welcome modifications or improvements by other developers to tailor the metadata management platform for different neuroscience initiatives.

## Methods

The metadata portal is designed to match the NeuroMorpho.Org metadata structure. Here first we summarize the organization of reconstruction metadata in this resource and then explain how the architectural design of the portal optimally serves the needs of the project.

### Organization of NeuroMorpho.Org metadata

NeuroMorpho.Org stores over 120,000 digital reconstructions of neuronal and glial morphology from nearly 650 independent laboratories and more than 1000 peer-reviewed articles. Each reconstruction is associated with detailed metadata across 25 dimensions thematically grouped into five different categories, namely *animal*, *anatomy*, *completeness*, *experiment*, and *source* [33].

The animal category specifies the subject of the study: species, strain, sex, weight, development stage, and age.

The anatomy category designates the brain region and cell type. Each of these two dimensions is hierarchically divided into three levels, from generic to specific: for instance, hippocampus/CA1/pyramidal layer and interneuron/basket cell/parvalbumin-expressing. Three considerations are especially important in this regard: first, additional information can be added in multiple entries at the third level. In the above example, the brain region could be further annotated as left and dorsal; and the cell type as fast-spiking and radially oriented. Second, the anatomical hierarchies are loosely rather than strictly organized since the specific details reported in (and relevant for) different studies vary considerably. If another paper describes the brain region of its dataset simply as dorsal hippocampus (without mentioning sub-area and layer), the concept “dorsal” would shift up to the second level. Third, both brain regions and cell types depend dramatically on the animal species, and most substantially diverge at the vertebrate vs. invertebrate taxa. Whenever possible, NeuroMorpho.Org follows the BrainInfo classification and NeuroNames terminology for vertebrates [10], and Virtual Fly Brain for invertebrates [31].

The completeness category provides details on the relative physical integrity of the reconstruction (accounting for tissue sectioning, partial staining, limited field of view, etc.), the structural domains included in the tracing (soma, axons, dendrites, undifferentiated neurites or glial processes), and the morphological attributes included or excluded from the measurement (most importantly, diameter and the depth coordinate).

The experiment category consists of methodological information describing the preparation protocol (e.g. in vivo, slice or culture), condition (control vs. lesioned, treated or transgenic), visualization label or stain, thickness and orientation of slicing or optical sections, objective type and magnification, tissue shrinkage and eventual corrections, and the tracing software.

The fifth category, source, provides details on the contributing laboratory, the reference publication, the original digital file formats, and the dates of receipt and release.

If any metadata dimension is not returned by the author or mentioned in the publication, the corresponding entry is marked as “Not reported” in the repository.

**Fig. 1:**
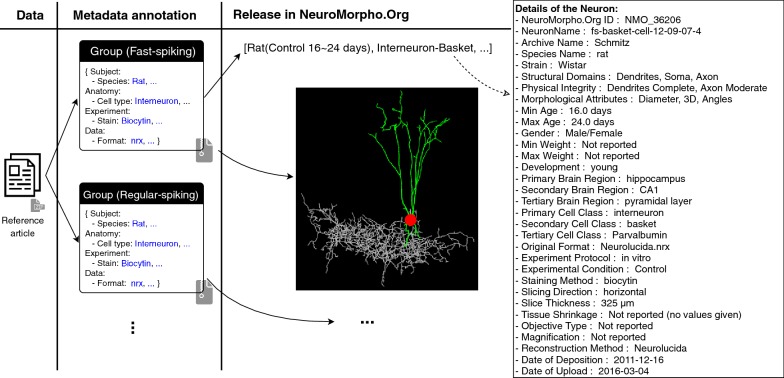
Metadata organization in NeuroMorpho.Org. Every dataset is associated with a publication and is typically divided into homogeneous annotation groups, each containing several reconstructed cells. Dots in the figure indicates continuation of groups and reconstructions. The formal database schema is publicly available at neuromorpho.org/images/Schema.png

Here we refer to ‘dataset’ as a collection of reconstructions associated with a single peer-reviewed publication. Many datasets are naturally divided into distinct metadata groups, either as a focus of the study (e.g. control vs. experimental condition) or because of cell-level specification of a particular variable (often animal sex or age). Typically, almost all metadata features are identical across the entire dataset except for specific details varying between groups. NeuroMorpho.Org preserves the same annotation organization at the levels of dataset, groups, and individual cells (Fig. 1). This intuitive yet compact structure conveniently allows both comparative statistical analyses and machine-readable accessibility via APIs.

### Design and implementation of the metadata portal

**Fig. 2:**
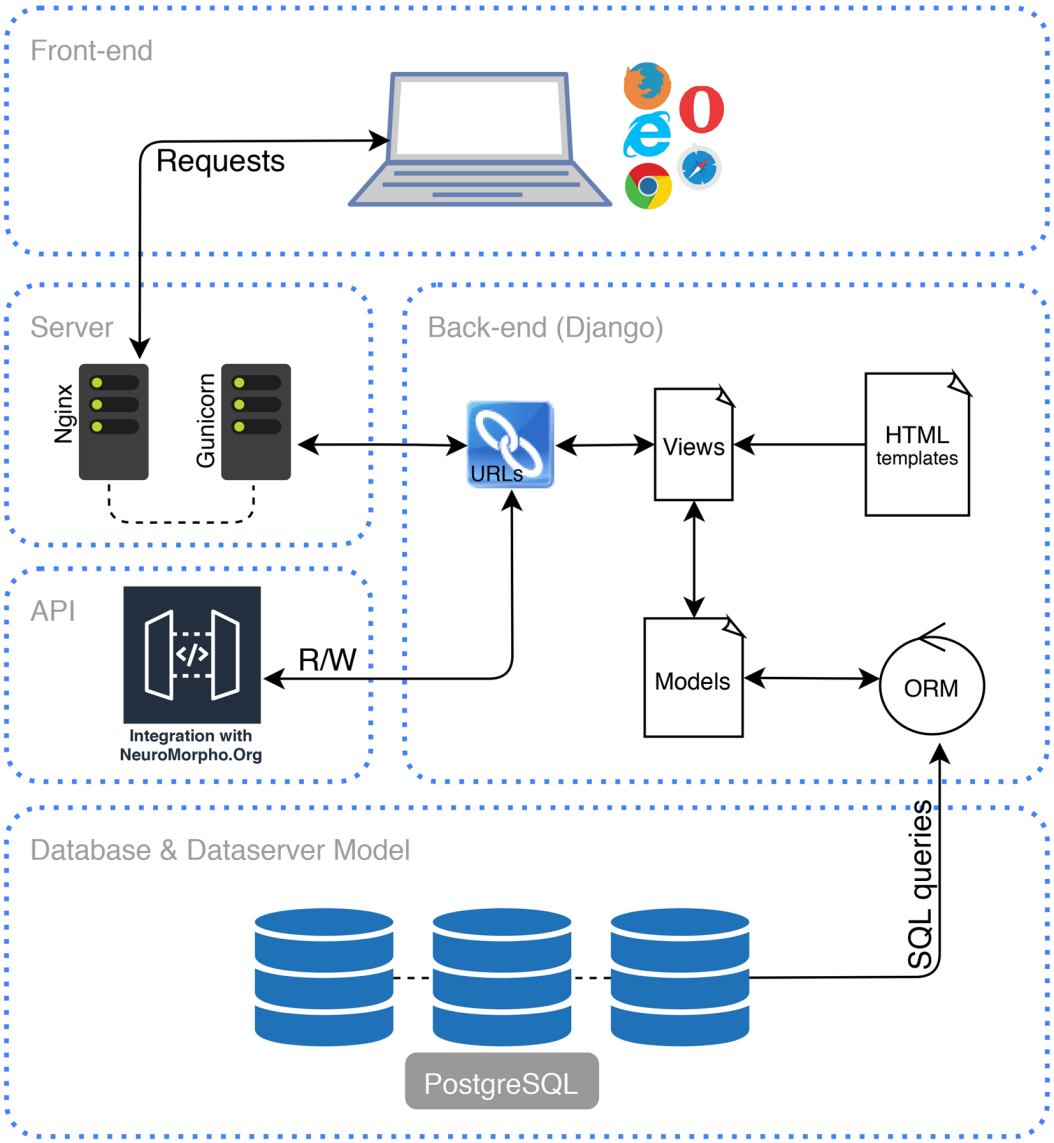
Overview of the system’s architecture. The code base of the metadata portal is running on Nginx and Gunicorn webservers. The Django controller handles all requests submitted by the users or received through the application programming interface (API), translates them into machine-readable commands and database queries, and returns the proper results

To ensure flexibility, scalability, portability, and efficiency, the metadata portal is designed based on the model-view-controller (MVC) software architecture [7]. This modular approach separates the application into three essentially independent components. The *model* represents the metadata structure and reflects the constraints, relations, and formats stored in the database through an object-relational mapper (ORM). The *view* defines the display presented to the operator through the graphical user interface (GUI). The *controller* mediates the requests of the user, interacts with the model, and generates an appropriate response for the view (Fig. 2). While anchoring the architectural foundation of the metadata portal onto a safe and trusted design pattern, the novelty of this development mostly lies in its goal and features that assist users in the metadata curation process.

The entire implementation abides by open-source principles and relies solely on open-source resources. The relational models of the portal in addition to the data are maintained in PostgreSQL, a fast, secure, and extensible relational database management system. The user interface is formulated by HTML, JavaScript, and Bootstrap, a Cascading Style Sheet (CSS) framework directed at responsive front-end web development. The control back-end is programmed in Django, a Python-based framework emphasizing pluggable and reusable elements, to regulate the interactions between database and users. Such modular yet integrated web-based framework offers rapid, cost-effective, and customizable application development. The resulting application is effortlessly accessible anytime across different platforms, enhancing interoperability and enabling different classes of users (authors, admins, and curators) to use the system independently while maintaining their work in the database.

The metadata portal encompasses most of the essential components to fulfill the curation needs of NeuroMorpho.Org. At the same time, it is also continuously evolving as new operational capabilities are prioritized. Recently developed features include: (i) the API (http://cng-nmo-meta.orc.gmu.edu/api/) enabling data interaction between the metadata portal and NeuroMorpho.Org; (ii) keyword search (http://cng-nmo-meta.orc.gmu.edu/search/), a user-friendly search engine allowing users to look for available terms in the database and their hierarchy; and (iii) bulk-modification feature, providing the ability to modify a large portion of terms within datasets.

The user interface of metadata portal offers seamless access to different parts and features of the system. The main page (http://cng-nmo-meta.orc.gmu.edu/) lists all active datasets. Each dataset is annotated with the name of the data contributor, publication identifiers (PMID and URL), and information regarding grant support. Metadata groups and their corresponding labels can be entered manually or are automatically created upon uploading grouped reconstruction files. Next, users select the actual entries for every metadata dimension, and the entire information remains accessible and editable through the web form. A detailed step-by-step metadata annotation protocol follows at the end of the Results.

## Results

We deployed the metadata portal for internal usage in the NeuroMorpho.Org curation team in spring 2018 after release v.7.4 of the database, which contained 86,893 reconstructions. The most recent release at the time of this writing (fall 2019), v.7.9, contains 121,578 reconstructions. Thus, we completed five full releases and annotated nearly 35,000 new reconstructions using the novel system described in this article. Moreover, we analyzed the records regarding metadata entry over four releases prior to deployment of the current system, namely, from right after release v7.0 (fall 2016), which contained 50,356 reconstructions. In the next section, we describe the positive impact on the project of switching from offline spreadsheet annotation to the web-based metadata portal.

### Metadata complexity, time saving, and error reduction

The metadata form in NeuroMorpho.Org employs more than 40 fields to encompass the details of the experiment, as several dimensions (e.g. animal weight and age) require more than one field (e.g. a numerical value and a unit scale). If treated as free text entry, many terms can be written in multiple equivalent variants, as in ‘mouse’, ‘Mouse’, ‘mice’, ‘mus musculus’ as well as being prone to semantically deviant typos (‘moose’). When considering the combination of all metadata fields, even in the absence of errors, the exact same information can be annotated in more than 10,000 distinct ways. Such an extreme case of combinatorial synonymy raises serious database management issues, in addition to slowing down search queries and requiring substantially inflated curation efforts. While the ‘mouse’ example may appear innocuous, even professional annotators can rapidly slide outside their zone of comfort when trying to distinguish between terminological equivalence and subtle but important differences in a genetic manipulation, staining process or electrophysiological firing pattern. The metadata portal offers a solution based on a corpus of controlled vocabularies consisting of public NeuroMorpho.Org content practically organized in user-friendly dropdown menus with autocomplete functionality and ‘similar hits’ suggestions. Moreover, the web form is endowed with hierarchical logic so that, for example, rat strains are not presented if mouse is selected as species.

Another major aspect of metadata annotation is the ongoing necessity to add new terms to describe previously unencountered entries. While certain dimensions, such as developmental stage, sex, objective type, and physical integrity, remain essentially unaltered over time, others, including brain regions, cell types, and experimental conditions, grow continuously at rates of approximately 5% (amounting to hundreds of new entries) per database release (Table 1). The web-based system facilitates the management of new concepts by enabling submission of free-text entries when needed; these are logged in real time into the database, allowing secondary review and provenance tracking.

**Table 1:**
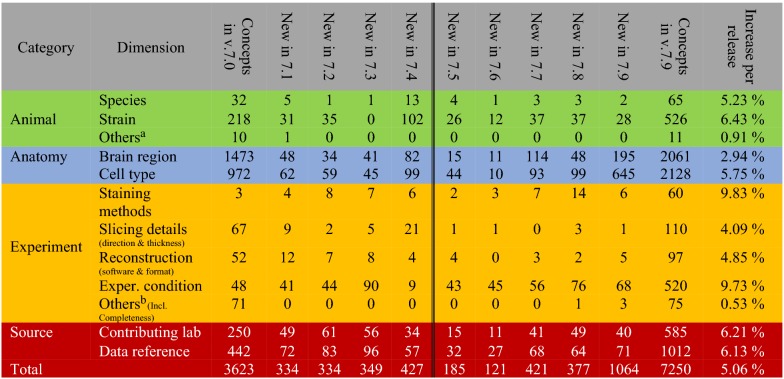
Number of distinct scientific concepts in the metadata portal arranged by category, with separate indication of newly added concepts in recent releases

7.x denotes version of the NeuroMorpho.Org. The vertical doubled line indicates the adoption of the Metadata Portal by the internal curation team

^a^Developmental stage, Sex (continuous variables Age and Weight are not relevant here)

^b^Protocol design, Objective type, Physical integrity, Structural domain, Morphological attributes (continuous variables Magnification and Tissue shrinkage are not relevant here)

Note that the growth of the data has maintained an approximately constant pace throughout the analyzed period, with similar amounts of metadata annotations considered before and after the introduction of the portal. Based on our lab records and analytics reports, the initial manual annotation of datasets in the last four releases (v.7.1–4) prior to deploying the metadata portal took an average of 1 h and 40 min per article (100 ± 10 min, mean ± standard deviation; N = 308 articles). The mean time required for the same operation in the five subsequent releases following the introduction of the portal (v. 7.5–9) dropped to 55 ± 5 min per article (N = 166), corresponding to a net saving of 45 min in the first step of metadata curation for each dataset. Moreover, all new terms need to be identified both to ensure appropriate database updating and synchronization, and to inform users upon release. This operation used to be carried out manually by visually inspecting each form, which normally required 14 ± 1 h of labor per release. The web-based portal automatically logs and reports all new terms, thus completely eliminating the need for this effort.

After the first annotation phase, metadata curation requires a second step of quality check after the preview release on the password-protected server and corresponding review by data contributors and database curators prior to public release. In most cases, this second phase entails at least some corrections and adjustments. When metadata was entered manually through a regular spreadsheet form (through v.7.4), most errors requiring corrections consisted of spelling mistakes (‘neocrotex’ instead of ‘neocortex’) or use of non-preferred terms (‘isocortex’ or ‘ctx’). A less common type of corrections involved conventional order of entries, as in “neocortex > medial prefrontal > right” vs. “neocortex > right > medial prefrontal”. Altogether, these issues required 100 ± 15 corrections per release in the old system. Use of controlled vocabularies, dropdown menus, smart filters, and autocomplete functionality dramatically reduced these instances to as few as 15 ± 5 per release. Corrections are especially taxing on data curators and database administrators, because mistaken ‘new’ entries need to be removed post-ingestion to avoid inconsistencies, indices and caches cleared, and synonyms properly linked for searches to work as intended. The drastic reduction in the number of required corrections saved about 18 h of labor per release, from 22 ± 3 prior to portal adoption to 4 ± 1 afterwards.

**Fig. 3:**
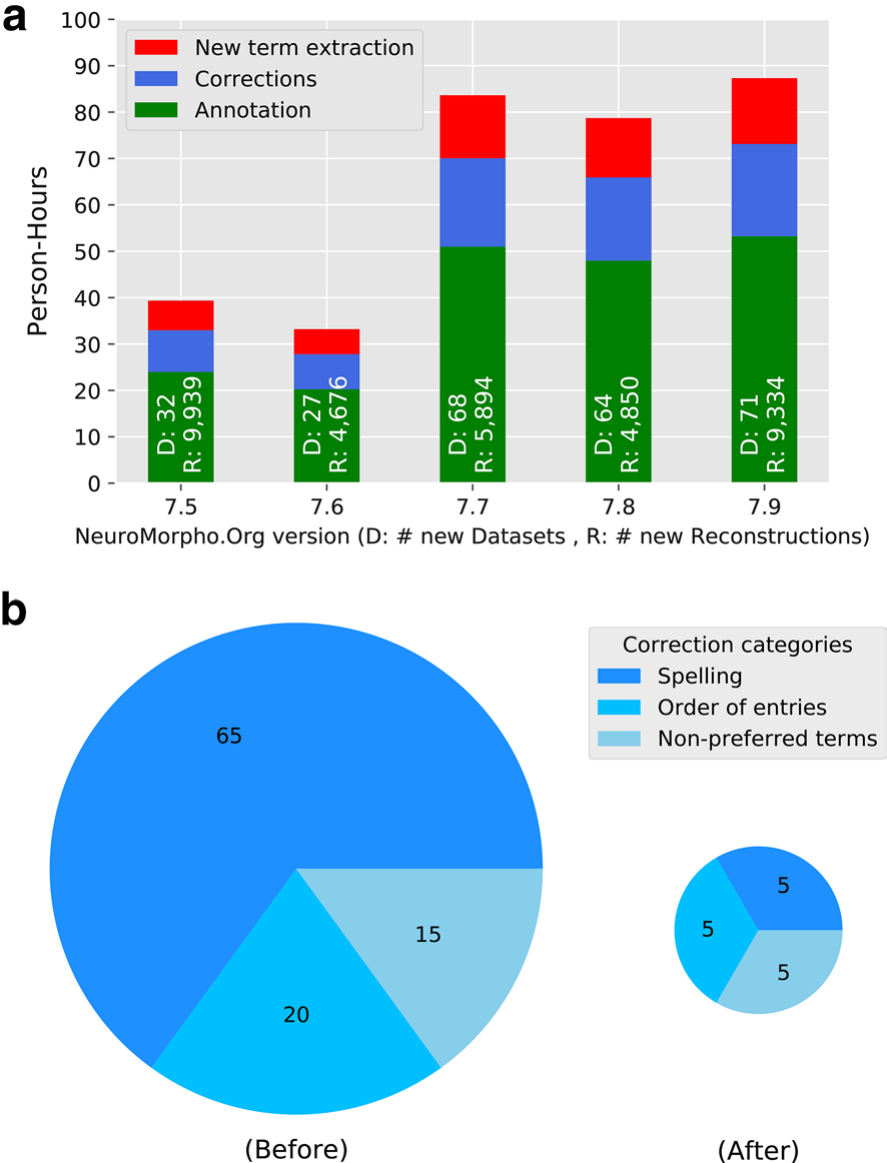
Labor-saving per version release yielded by the metadata portal. **a** Effort saved by the adoption of web-based annotation in the last 5 releases (7.5 to 7.9) of NeuroMorpho.Org. **b** Detailed categories of annotation mistakes requiring post-ingestion corrections during the review phase before (left) and after (right) transitioning to the new annotation system; the average numbers of necessary corrections per release are indicated inside the pie charts

When considering all sources of time saving (annotation, new term extraction, and corrections), the introduction of the web portal reduced the metadata annotation effort from 115.6 ± 35.4 to 48.3 ± 19.5 person-hour/release, a 58% effort reduction (Fig. 3).

### Usage protocol

In addition to the many advantages of the metadata portal described above, the web-based implementation naturally enables its direct usage by the authors of the articles described the original datasets, namely the data contributors. Considering the greatly improved performance of metadata annotation, with this article we invite all researchers depositing their neuronal and glial tracings into NeuroMorpho.Org to utilize the portal for annotating their submission. In this section, we overview the functionality, features and usage of the system http://cng-nmo-meta.orc.gmu.edu/.

**Fig. 4:**
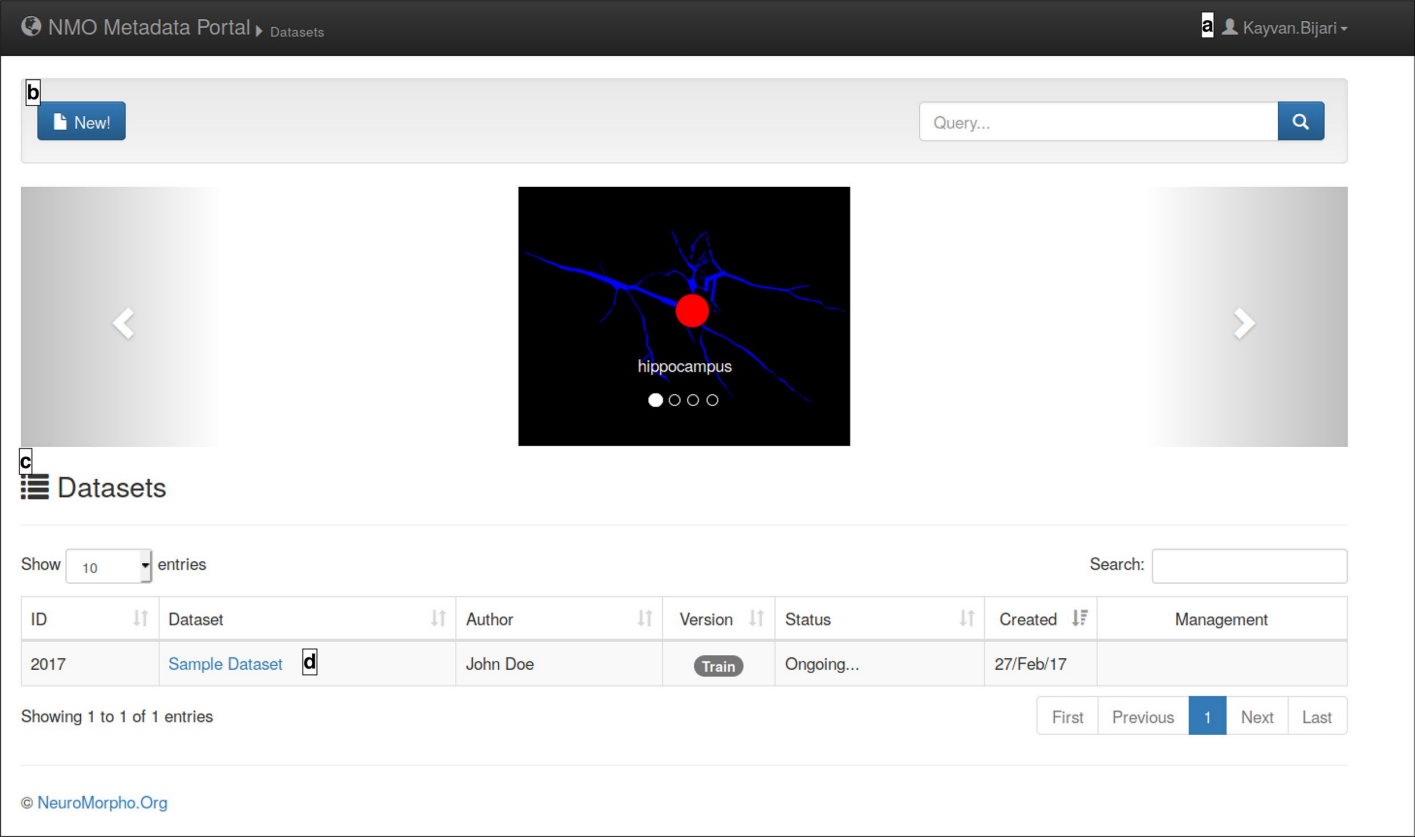
View of the portal’s main page. **a** Logged-in user. **b** ‘New!’ button to create a dataset. **c** Table listing the available datasets for the user in the system. **d** Sample (demo) dataset

In order to limit the server susceptibility to automated malicious activities, users must log in via username (nmo-author) and password (neuromorpho) or using a Google account. Using the latter approach, the user’s entry remains private (only visible to the contributor and the administrators, but not to other users) until approved for public release by the NeuroMorpho.Org curators. Upon entering the portal (Fig. 4), users can create a dataset by clicking on the ‘New!’ button in the main view.

**Fig. 5:**
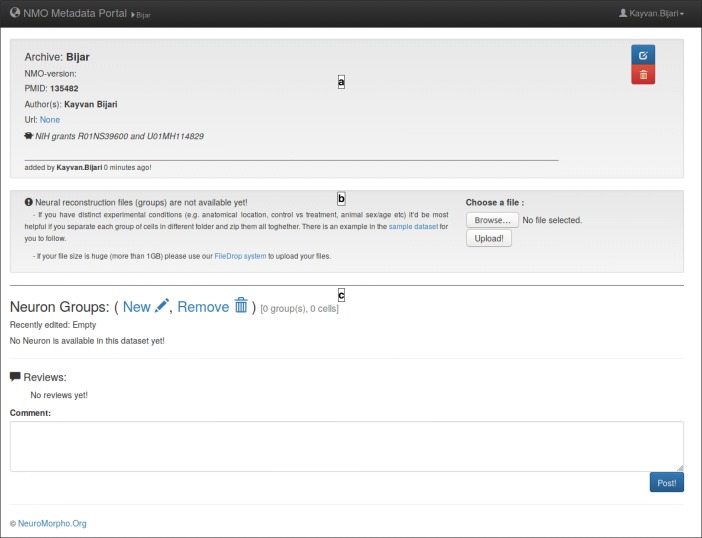
Newly created dataset in the metadata portal. **a** Basic information about the dataset as well as edit and remove buttons. **b** Reconstruction section to upload digital tracing files. **c** Menu for creating, editing, and removing the experimental groups associated with the dataset

The newly opened window prompts the insertion of information related to the reference publication such as PMID, authorship, and grant support. Next, clicking ‘Submit & create the dataset’ transitions to the next phase, namely uploading reconstruction files and defining the experimental groups (Fig. 5).

**Fig. 6:**
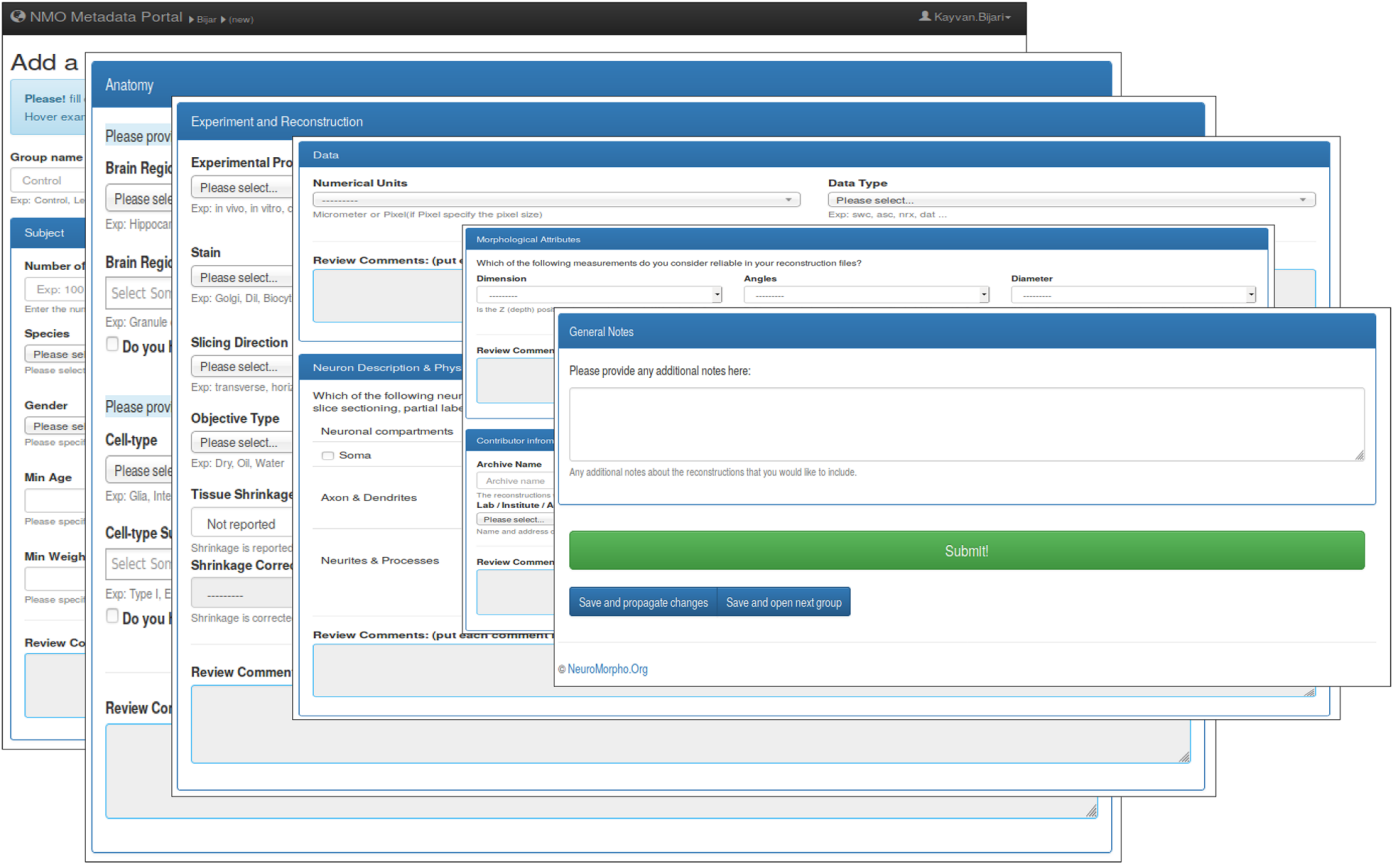
Metadata form to annotate the details of the reconstruction within each experimental group

To upload reconstruction files, users should click the ‘Browse’ button to locate the zip folder containing the data. Separate groups with distinct experimental conditions (control vs. treatment, but also different anatomical locations, animal sex/age, etc.) must be organized as corresponding folder in the compressed archive. The ‘New’ button in the Neuron group section adds an experimental group and calls a new form window requesting the corresponding metadata details (Fig. 6).

**Fig. 7:**
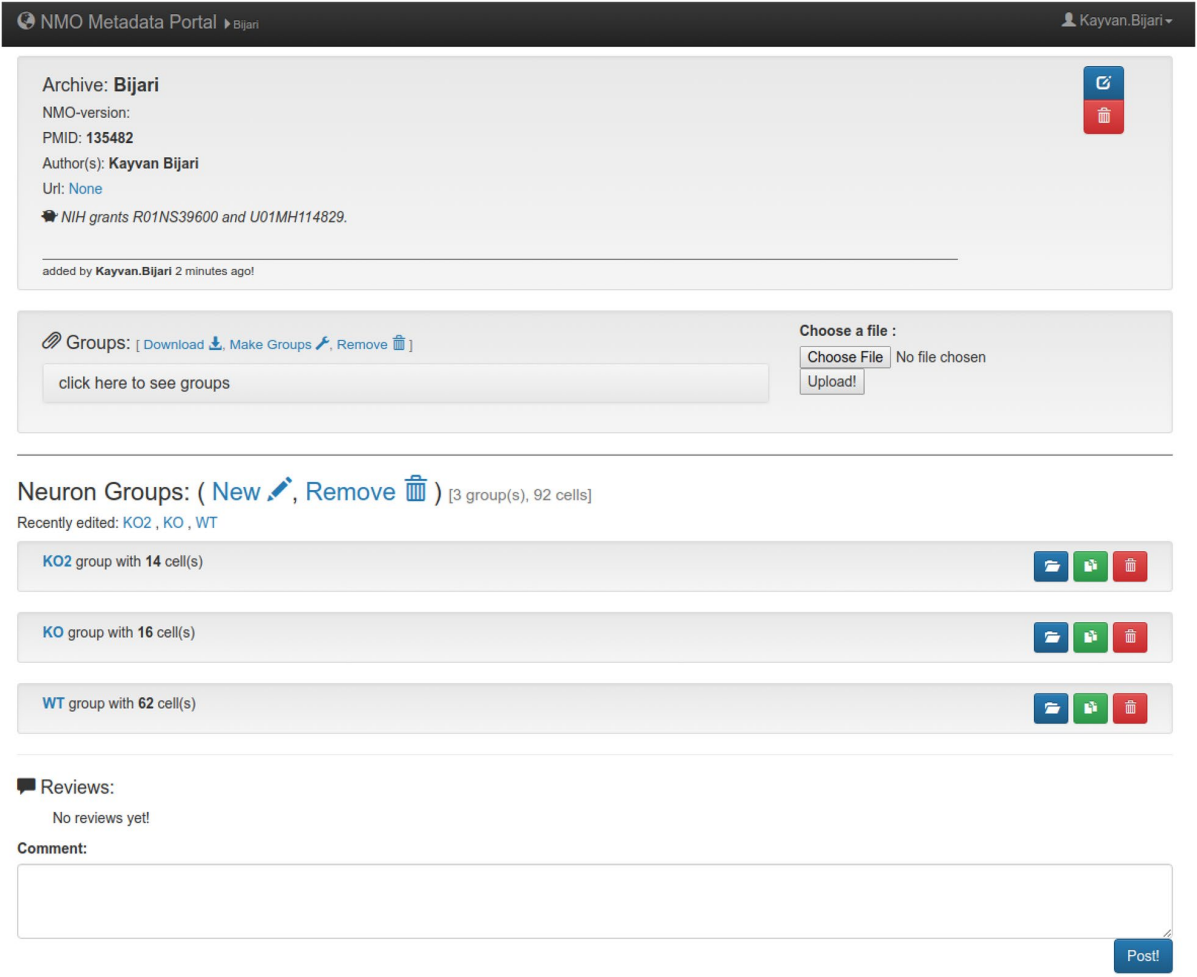
Final dataset with reconstructions and all experimental groups added in the metadata portal

After filling out the entries as completely as possible, the user can click on ‘submit the group’. In case of multiple groups, the auxiliary buttons facilitate duplication, propagation, and modification of metadata details (Fig. 7).

Shortly after final submission, the internal NeuroMorpho.Org secondary curation begins, which includes validating the newly added terms. The reconstruction files along with the descriptive metadata are then ready for ingestion and release on a password-protected preview site that mirrors the look-and-feel of NeuroMorpho.Org while allowing extensive review of content, annotations, and functionality by data contributors and curators prior to public release.

## Conclusion

Continuous growth of neuroscience knowledge requires a parallel maturation of informatics resources to annotate data for future re-use and interpretation. This report introduced a newly developed metadata portal that leverages web-based technologies to facilitate effective curation of digital reconstructions of neuronal and glial morphologies. All components of this framework are open-source and can thus be adopted for or adapted to the needs of other related projects. Moreover, the metadata portal is ready to be integrated with artificial intelligence modules such as natural language processing or smart recommendation systems to further expedite and improve the critical bottleneck of database curation. Recently, machine learning algorithms have been successfully deployed for metadata extraction [27]. In particular, text mining tools, such as *named entity recognition*, can learn, identify, and label crucial elements of neuroscience documents like neuron names, brain regions, and experimental conditions [5, 37] . Hence, our future aim will be, first, to train and validate a model on the growing set of curated articles in the NeuroMorpho.Org literature database, as well as on the named entities therein; and then to deploy it on the metadata portal in order to facilitate assisted keyword extraction. To be clear, we consider it unrealistic to expect full automation of all metadata extraction tasks in the near future, as too many decisions involve domain-specific expertise and often ad-hoc conventions. Nevertheless, the prospect of a hybrid human–computer interface ergonomically optimized to maximize the breadth, depth, and accuracy of annotation while minimizing time and labor is in our view well within reach. As a first step in that direction, the systematic coding of the prior entirely manual spreadsheet annotation process of NeuroMorpho.Org metadata within a web-form interfaced to a back-end database has already substantially reduced the ongoing curation effort. We are now releasing this system publicly to allow willing data contributors to enter the details of their datasets directly at the time of data submission. While the design of the portal still allows and encourages an iterative process of collaborative review to reduce the risk of ambiguity and inconsistencies, we hope that enabling metadata annotation by the “ultimate experts” who produced the data will bring us closer to a robust, distributed, and dynamic community-based resource.

## Data Availability

Project name: NeuroMorpho.Org Metadata Annotation. Project home page: http://cng-nmo-meta.orc.gmu.edu/. Operating system: Platform independent. Programming language: Python, HTML, Java script. Other requirements: Python 2.7, Django 1.9, Nginx License: GPL 3.0. Source code: https://github.com/NeuroMorpho/metadata-portal.

## Abbreviations

API: Application programming interface
CMS: Content management system
HTML: Hyper text markup language
NIF: Neuroscience information framework
OWL: Web ontology language
PDF: Portable document format
REST: Representational state transfer
RW: Read and write
SQL: Structured query language
UI: User interface
URL: Uniform resource locator
XML: eXtensible markup language
